# Genetic Information to Share with Parents when Newborn Screening Reveals the Presence of Sickle Cell Trait

**DOI:** 10.1155/2024/8910397

**Published:** 2024-02-22

**Authors:** Narcisse Elenga

**Affiliations:** ^1^Pediatric Medicine and Surgery, Hôpital Andrée Rosemon, Rue des flamboyants, BP 6006, 97306 Cayenne Cedex, French Guiana; ^2^Sickle Cell Reference Center, Pediatric Medicine and Surgery, Centre Hospitalier de Cayenne “Andrée Rosemon” Rue de Flamboyants, BP 6006, 97306 Cayenne Cedex, French Guiana

## Abstract

The primary purpose of newborn screening for sickle cell disease is to diagnose the disease before the appearance of symptoms and to initiate early treatment. To answer the question “What genetic information needs to be communicated to parents when newborn screening reveals the presence of a sickle cell trait,” we conducted a survey using a self-administered online questionnaire. We received responses from 122 healthcare workers and members of sickle cell disease associations, in France and French overseas departments. Our results showed similar positions on this issue. The information conveyed is not consistent and is the result of grassroots initiatives. The negative consequences generated by this information could be reduced when this information is delivered by a multidisciplinary team, within the framework of a dedicated consultation. This information on sickle cell trait status should be given in at least three key periods: the neonatal period, early adolescence, and later adolescence, when reproductive implications become important. Neonatal screening programs should develop systems that allow referring physicians to easily access the results of neonatal screening electronically. Harmonization of practices should allow a better analysis of the consequences of this counselling on family projects.

## 1. Introduction

First described in 1910 [1, 2], sickle cell disease (SCD) is also called hemoglobinosis S or sickle cell anaemia. It is an autosomal recessive inherited disease affecting the hemoglobin in red blood cells. SCD affects more than 50 million people worldwide, making it the most common genetic disease in the world [3]. In people with this disease, there is an abnormal hemoglobin: hemoglobin S, due to the substitution of valine by glutamic acid in the beta chain of globin. When the oxygen concentration in the blood decreases, the red blood cells become deformed and take on the shape of sickles instead of being biconcave. This results in several characteristic symptoms of the disease, the most common of which are chronic anaemia, painful vasoocclusive crises (VOC), and increased susceptibility to infection. Heterozygous subjects are the healthy carriers of hemoglobin S. They are clinically asymptomatic, but two heterozygotes can give birth to sick children. This disease appeared independently in Africa and India and particularly affects the populations of these regions. However, population movements have also made it very present in America, especially in the Caribbean and Brazil, and in Western Europe [3]. In France, 431 sickle cell children and 12619 heterozygotes were born in 2016, i.e., in metropolitan France, an incidence of one child with major sickle cell syndrome (MSC) for 823 (IC95% 1/918-1/745) newborns tested for sickle cell disease in a targeted manner and 1/2088 (IC95%1/2330-1/1892) of all births. This makes it the most common genetic disease in France. However, this incidence is much higher in the French overseas departments (1/525 (IC95%1/678-1/428)) and in the Paris region (1/824 (IC95% 1/950-1/727)) where the populations at risk are concentrated [4]. There is heterogeneity in this incidence in metropolitan France (it is higher in the Ile de France) but also between the French overseas territories (French Guiana/Reunion). The incidence in French Guiana is the highest of all French territories, i.e., 1/206 (IC95% 1/307-1/154), and the lowest in Reunion Island, with 1/1968 (IC95%1/7586-1/1130).

Since 1995, the French Association for the Screening and Prevention of Childhood Handicaps (AFDPHE), taking into account preliminary experiences, has organized and implemented a national neonatal screening program (NSP) for SCD. This screening was generalized in metropolitan France in 2000. It is systematically performed in children of parents from the populations most affected by the disease. In the French overseas territories, this screening is offered to all newborns. For affected children, it allows the earliest possible start of preventive treatment for anaemia and infectious complications. The screening is biochemical, and unlike genetic screening, there is no written consent but an oral consent with two specific information documents “sickle cell syndrome major and heterozygous” with the name of the attending physician concerned [5–7]. The advantage of reporting carriers of sickle cell disease is the detection of a greater number of at-risk couples (whose two parents are carriers) who can be informed of their future reproductive choices, a responsibility that falls to their general practitioner. Thus, the AFDPHE has developed brochures and other information materials relating to neonatal screening, aimed at both health professionals and families. In the case of a healthy carrier child, how should this information be given to the parents, knowing that the information related to the screening concerns a diagnosis of disease and not information on genetic status? For parents of a child with a SCD trait, genetic counselling is a crucial step in preventing this serious, potentially fatal condition and in informing them of the health risks associated with it. In fact, communication of positive newborn screening results for sickle cell trait varies from country to country and, within countries, from hospital to hospital. However, there is very little literature in this area. In addition, neonatal screening for hemoglobin abnormalities in the population raises unresolved questions for which there are no recommendations or consensus. Some centres advocate joint screening of newborns and their parents. However, the involvement of screened children and adolescents remains a pertinent issue, given the persistent perception of the trait as a pseudodisease and the risk of reifying a biological difference between relatives. Others recommend a letter followed by a phone call or text message. In order to improve the prevention of sickle cell anaemia, we need to raise public awareness and train general healthcare professionals, involving the media and schools.

The main objective of this study was to explore the appropriateness and method of returning information to families when a healthy hemoglobin S carrier infant is screened at birth. To explore this question, we chose to rely on an online self-administered questionnaire intended for health professionals and members of sickle cell disease associations. The secondary objective was to discuss how to improve this information and its content to reduce gaps in reporting of maternal and infant sickle cell trait outcomes and to understand and promote informed disclosure decisions.

## 2. Methodology

### 2.1. Questionnaire

A 19-question questionnaire was developed taking into account the objectives of the study (see the appendix). The content of the questionnaire was the modalities of the announcement of the diagnosis and the reactions of the parents that could be expected once the diagnosis was announced. Among these questions, there were 18 closed multiple choice questions and one open question. The average time to complete the questionnaire was estimated at 10-15 minutes.

### 2.2. Population Concerned

This self-administered questionnaire was intended for health professionals in reference and competence centres for major sickle cell syndromes and for members of associations for the fight against sickle cell disease (some of whom were parents of children with hemoglobin S), in metropolitan France and in the French overseas departments.

### 2.3. Distribution of the Questionnaire

Information on the existence of this questionnaire was made available online by the coordinator of the healthcare network Constitutional Diseases of the Red Blood Cell and Erythropoiesis (MCGRE), after authorization from its coordinator. The members of the network were asked to answer the questionnaire on several occasions, through the secretariats of the patient associations and by direct solicitation for the health professionals in the network.

### 2.4. Administration of the Questionnaire

The questionnaire was self-administered online (using the free online survey software SurveyMonkey), by the MCGRE health network platform to all its members, i.e., 600 people.

The sample consisted of 122 respondents. The questionnaire was administered on June 02, 2016.

### 2.5. Expertise of Interviewees

The people interviewed had expertise in the field, such as physicians involved in the management of SCD, some of whom had expertise in neonatal diagnosis of SCD, a national referral biologist for neonatal screening for SCD, a social psychologist and psychotherapist, a representative of associations fighting against SCD, and a clinical psychologist, member of 3 associations: a member of the board of directors of Rofsed, Drépan'ose, and URACA-Basiliade with extensive experience in the announcement of neonatal diagnosis.

### 2.6. Data Analysis

The aim of our analysis was to describe the responses of the interviewees on the relevance of screening heterozygous newborns, the time of the announcement of the result, the support and content of the information given, and the place and duration of the interview allowing this information.

## 3. Results

### 3.1. Persons Surveyed

Of those who responded to the questionnaire, 72% were physicians and 9% were nurses. Members of associations for the fight against sickle cell disease were 4% who answered the questionnaire.

### 3.2. Modalities of the Announcement of the Diagnosis of Heterozygosity

54% of the respondents stated that, in their centre, the announcement of the diagnosis of heterozygosity is made during a regular medical consultation. In 15.5% of cases, this announcement was made during a genetic counselling consultation. On the other hand, in 24% of cases, the announcement was made only by mail. 57% of the respondents stated that genetic counselling was available in their centre. Genetic counselling was performed in the presence of either a genetic counsellor (89%) and/or a clinical psychologist (35%). The majority of respondents (>95%) thought that the counselling interview should be conducted by a multidisciplinary team led by a physician with expertise in sickle cell disease.

### 3.3. Arguments for and against Screening Heterozygotes

When asked “should heterozygotes be screened?”, 93% said yes, stating that it was of value for genetic counselling. The vast majority (98%) said that the purpose of newborn screening for sickle cell disease was to screen newborns with major sickle cell syndrome. 50% felt that parents should be asked if they would like to be informed of the screening result, in the newborn period, in the case of heterozygosity. 47% of the participants said that it is obvious that parents should be informed regardless of the result. 60% thought that there was a risk of loss of vision by delaying the announcement of the result. 27% said that since there are no clinical manifestations, no immediate therapeutic sanction, for such an anxiety-provoking diagnosis, there was no risk in informing parents later.

In addition, the information gets lost and would need to be rephrased later. In all cases, the majority of respondents (96%) thought that parents should be informed of the results of screening even if the child is only heterozygous or a healthy carrier. The reasons given were multiple and can be summarised as “this child in adulthood should take this into account when choosing a spouse,” “to inform them of the existence of the gene in their family and of the possible risks of the occurrence of major sickle cell syndrome,” “to screen for the risks of both parents of having a homozygous child, and for the future of the child, so that he knows what he is at risk of transmitting,” and “question of access to medical information, of medical ethics, it is legitimate to know. Not informing puts the medical profession in a difficult position”.

### 3.4. Content of the Information Letter or Message

Concerning the content of the information letter to be delivered to the parents, half of the people interviewed recommended a standardised, detailed message.

### 3.5. Duration of the Interview

For almost 90% of the respondents, the duration of the interview with the parents should be less than one hour.

The reactions of the parents to the information about the heterozygotes are summarised in Table 1.

Table 2 summarises the responses to the question, “How can parents' concerns about being told they are heterozygous be addressed?”

The people interviewed stated that the reactions of the parents following the announcement are diverse and varied: significant emotional charge, incomprehension, fear, stigmatisation, flabbergasted, feeling of a relentless fate, and anxiety.

### 3.6. Answers to Open Questions

The other issues raised by the respondents can be summarised in 5 points:

- Not informing poses an ethical problem: Article R.1131-5 from the decree of June 23, 2000, bioethics law of August 6, 2004
- Free and informed consent or information before screening is necessary
- Communication should be improved in order to reduce parents' anxiety and to allow a better understanding of the heterozygous status, in order to avoid confusion
- It should be recognized that it is already very difficult and time-consuming to announce a major sickle cell syndrome, but this is essential for subsequent management. The announcement of heterozygosity would seem to be even more time-consuming (large number of patients) and very difficult to understand
- Knowledge of parental status should perhaps be dissociated from knowledge of the heterozygous status of the newborn

Finally, a physician trained in the management of sickle cell disease should conduct the interview, according to the majority of respondents (Table 3).

We can summarise the areas and the number of questions per area: timing of the announcement (40% during a regular medical consultation) support and content of the information given, location, and duration of the interview; respondents answered about how things are currently done or how they should be done.

## 4. Discussion

The primary objective of neonatal screening for sickle cell disease is not to identify the sickle cell trait. However, the tests used inevitably identify heterozygosity much more frequently than major sickle cell syndromes. Moreover, this discovery raises many questions, in particular that of the information to be provided about this condition. The people surveyed had similar positions on this issue. Thus, the question of screening, particularly for newborns of parents from the most at-risk populations, no longer arises [8–10]. In fact, nearly 90% of the people interviewed in this survey stated that it was useful to screen heterozygotes. Indeed, the eventual discovery of heterozygosity in a newborn is inextricably linked to neonatal screening for sickle cell disease.

Information to the parents of a heterozygous newborn is the result of field initiatives and is unequally practiced in France [11]. It is indeed a complex information which not only makes parents anxious but also can be associated with questions and misunderstandings about the consequences both on health and in terms of family projects. In addition, there is a risk of permanent confusion between “abnormal” and “heterozygous.” The majority of the respondents affirmed that the announcement interview should be conducted by a multidisciplinary team led by a doctor with expertise in sickle cell disease. Half of these people advocated a standardised but detailed and clear message. However, when we see the high annual number of heterozygous newborns in France, this announcement poses a real medico-economic problem. Indeed, the centres of reference are already overloaded and have no budget or additional staff to fully carry out this task. Parents' reactions following the announcement are diverse and varied, ranging from concern to anxiety. It will therefore require a great deal of communication effort, so as not to create excessive anxiety in the parents, again creating a heavy workload in terms of genetic counselling [12]. And above all, according to the majority of the respondents, it is to avoid the telephone call to announce bad news.

The majority of the respondents agreed with the communication of the result at birth. Sixty percent thought that there was a risk of losing sight of the family by delaying the announcement. On the other hand, almost a third thought that since there are no clinical manifestations, no immediate therapeutic sanction, for such an anxiety-provoking diagnosis, there was no risk in informing the parents later. Moreover, the information is lost and would have to be rephrased later.

For the majority of respondents, free and informed consent or information, integrating the notion of a healthy carrier before screening, is necessary. Is it possible in the current state of knowledge to dissociate the screening of the disease from the screening of the heterozygous state? Today, new screening techniques are appearing such as mass spectrometry already used in the United Kingdom and in the Lille screening laboratory. This methodology requires sophisticated data analysis software that can be parameterized so that heterozygotes are ignored. This was the choice made in Wales [13]. Does it make sense to establish two separate consents? The first concerns the diagnosis of a disease, sickle cell anaemia, in the newborn. The second would concern the parents' agreement to the communication of genetic information, obtained during this diagnostic process in a newborn child not affected by the disease, but which may be of interest for a future child. Given that this information on a child carrying the sickle cell trait should be given one day, according to the opinion of the overwhelming majority of the people surveyed, this approach could be counterproductive, especially in regions with a high incidence of sickle cell disease. It should be remembered that we are all likely to be carriers of several mutations corresponding to recessive diseases. The notion of beneficial or harmful mutation can vary according to the environment and many other factors. For example, the notion of heterozygosity for a gene, when it does not in itself cause disease (which is the case for almost all recessive genetic diseases) may not only be neutral for health but confer a benefit in certain environments [14]. On the other hand, if parents do not want to know their genetic status, the opportunity to screen at-risk couples will be missed and genetic counselling will not be available. For the newborn, keeping this information secret for 20 years could lead to a risk of losing the information (this information is often noted in the child's health record). On the other hand, as revealed by the majority of respondents, leaving a family in the dark poses an ethical problem with regard to the possible search for the mutation in both parents [15, 16], which, if positive (which will not be the case in more than 90% of the cases), could lead to a prenatal or preimplantation diagnosis. Between ignorance and concern, we must above all question the conditions and the moment when access to such information should be the most useful. In the United States, despite generalized neonatal screening for sickle cell disease, only 37% [17] of parents are informed when their child is a carrier of the sickle cell trait. Furthermore, this information is diluted over time to the point where only 16% [18] of individuals of childbearing age who carry the sickle cell trait have this information. In addition, patients may not have the same treating physician between birth and adolescence. This information about sickle cell trait carrier status should be given at at least three key periods: the neonatal period, to inform parents about the risk that they may have of conceiving a sickle cell child in the future; early adolescence, when strenuous exercise may be initiated (with the risk of exceptional sickle cell disease); and later adolescence, when the reproductive implications become important. Neonatal screening programs should develop systems that allow referring physicians easy access to electronic neonatal screening results [19].

Adolescence could thus be an opportunity for a reminder not to be missed. But on what occasion? If the result of the hemoglobin electrophoresis was already recorded in the health record, the reminder of this information could be made when registering for sports activities, by asking for a review of the record. At this age, the child has enough ability to seek out information on what it means to be a carrier of the sickle cell trait [20, 21].

Our study has some limitations: while the questionnaire targeted both health professionals and members of associations, including parents of heterozygous children, 81% of the responses came from doctors and nurses, which does not allow us to study whether professionals and patients have a different opinion. Furthermore, the percentage of informed parents who actually arrive at the consultation is not known with accuracy.

## 5. Conclusion

The discovery of heterozygous status during newborn screening for sickle cell disease raises many questions about how to deliver information about this condition. The people interviewed had similar positions on this issue. The negative consequences generated by this information could be reduced when this information is delivered by a multidisciplinary team, in the context of a dedicated consultation. This information on sickle cell trait carrier status should be given at at least three key periods: the neonatal period, early adolescence, and later adolescence, when reproductive implications become important. Neonatal screening programs should develop systems that allow referring physicians to easily access neonatal screening results electronically.

A harmonization of practices should allow a better analysis of the consequences of this information on family projects, thus making it possible to compensate for the absence of French data on the subject.

## Figures and Tables

**Table 1:** Parents' reactions to the diagnosis.

Parents' reactions	Numbers	%
High emotional burden	76	65
Incomprehension	85	72.7
Fear	82	70
Stigma	37	31.6
Dismay	23	20
Feeling of an implacable destiny	32	27.4
Anxiety	94	80.3
Total number of participants: 117	5 questions not answered	

**Table 2:** How to reduce parental anxiety at the time of the announcement?

How can parents' concerns be addressed?	Number	%
Avoiding the phone call to announce “bad news”	52	44
Standardised, short, precise message	34	28.8
Do not dramatise, but reassure	82	69.5
Do not give details about the illness (because the child is not sick)	27	22.9
Answer all parents' questions	114	96.6
Record the child's status in the health record	75	63.6
Total number of participants: 118	4 questions not answered	

**Table 3:** Who should do this interview?

Who should do this interview?	Numbers	%
Geneticist (physician or engineer)	45	38
Physician trained in the management of sickle cell disease	116	98
Clinical psychologist, trained in sickle cell disease	41	35
Other health professional trained in the management of sickle cell disease	55	47
Member of a patient association, trained in sickle cell disease	27	23
Total number of participants: 118	4 questions not answered	

## Data Availability

The data will be made available upon reasonable request.

## Appendix

### A. DREPAHETERO

Sickle Cell Disease Questionnaire

Post-screening questionnaire for health professionals and members of sickle cell associations (metropolitan France and overseas departments).

It asks for your opinion on the information given to parents of newborns who have been screened as heterozygous or healthy carriers. It does not concern sick newborns.

1. Who are you?

 A person with severe sickle cell disease

 A parent of a child with severe sickle cell disease

 A person who does not have sickle cell anaemia and is a member of a sickle cell anaemia association

 A doctor

 State-registered nurse

 State-registered specialist nurse

 Nursing assistant

 Nursery assistant

 Clinical psychologist

 Physiotherapist

 Social worker

Other (please specify)

2. How is heterozygosity often diagnosed in your centre?

 Ordinary medical consultation

 Genetic counselling

 Ad hoc multidisciplinary consultation

 By post

 By telephone

 Do not know

3. Does your centre offer genetic counselling?

 Yes/No

 Does not

 Do not know

4. If yes, with whom?

 A genetic counsellor

 A psychologist

 Other (please specify)

5. Do you think heterozygotes should be screened?

 Yes, why?

 No, why not?

 Do not know

6. What is the main aim of newborn screening for sickle cell disease?

 Screening of newborns with sickle cell disease

 Screening of heterozygous newborns (healthy carriers)

 Do not know

7. Should parents be asked if they wish to be informed about the results of screening during the neonatal period?

 Yes, why?

 No, why not?

 Do not know

8. Should parents be asked if they would like to be informed later?

 Yes, why?

 No, why not?

 No, why not?

9. Should parents be asked if they do not wish to be informed at all?

 Yes, why not?

 No, why not?

 No, why not?

10. Should parents be informed of the results if the newborn is heterozygous (a healthy carrier)?

 Yes, why?

 No, why not?

 Do not know.

11. Should parents not be informed of the results if the newborn is heterozygous (a healthy carrier)?

 Yes, why not?

 No, why not?

 Do not know.

12. What do you think should be the content of the information letter sent to the parents of a heterozygous or healthy carrier newborn (multiple answers possible)?

 Standardised message, short, no details

 Standardised, full, detailed message

 Non-standardised message, based on parents' questions

 Non-standardised message, based on parents' questions

 Other suggestion (please specify)

13. What do you think about the verbal content of the information to be given to the parents of a heterozygous or healthy carrier newborn (multiple answers possible)?

 Standardised message, short, no details

 Standardised, full, detailed message

 Non-standardised message, based on parents' questions

 Non-standardised message, answering parents' questions

 Other suggestion (please specify)

14. When talking to parents who already have at least one child with severe sickle cell disease, how can parents' concerns about a heterozygous newborn be allayed (multiple answers possible)?

 Avoid calling to give “bad news”

 Standardised, short, precise message

 Do not dramatise, but reassure

 Do not give details of the illness (because the child is not ill)

 Answer all the parents' questions

 Record the child's condition in the health record

 Other (please specify)

15. In the case of parents who do not have a child with severe sickle cell disease, how can parents' concerns about a heterozygous newborn be allayed during the interview (multiple answers possible)?

 Avoid calling to give “bad news”

 Standardised, short, precise message

 Do not dramatise, but reassure

 Do not give details of the illness (because the child is not actually ill)

 Answer all the parents' questions

 Record the child's status in the health record

 Other suggestions (please specify)

16. How long should this interview take?

 30 minutes

 30-60 minutes

 60 minutes

 >60 minutes

 Other suggestion, please specify

17. Who should conduct this interview (more than one answer possible)?

 Geneticist (doctor or engineer)

 Doctor trained in sickle cell disease management

 Clinical psychologist trained in sickle cell disease

 Other health professional trained in sickle cell disease management

 Member of a patient association trained in sickle cell disease

18. What kind of reactions can you expect from parents (more than one answer possible)?

 Heavy emotional burden

 Lack of understanding

 Fear

 Stigmatisation

 Dismay

 Feeling of fate's relentlessness

 Anxiety

 Other reactions, please specify

19. What other issues do you think the discovery of heterozygosity in a newborn raises?

 Finalised
